# Secukinumab-Induced Severe Palmoplantar Psoriasis treated with Guselkumab: A Case Report

**DOI:** 10.31138/mjr.110525.ary

**Published:** 2025-06-30

**Authors:** Ibrahim Al-Homood, Nourah Alaboon, Hoda E. Draz

**Affiliations:** 1 Department of Internal Medicine, Rheumatology Section, KFMC, Riyadh, KSA,; 2 KFMC, Riyadh, KSA

**Keywords:** secukinumab, palmoplantar psoriasis, interleukin, guselkumab

## Abstract

**Background:**

Secukinumab is a monoclonal antibody targeting interleukin-17A (IL-17A), approved for the treatment of moderate-to-severe plaque psoriasis and psoriatic arthritis. While effective for many patients, it has been associated with paradoxical reactions such as exacerbation of psoriasis or drug-induced dermatitis. This case report presents a unique occurrence of severe palmoplantar psoriasis triggered by Secukinumab in a patient with psoriatic arthritis and multiple sclerosis (MS), necessitating a switch to another biologic therapy.

**Case Presentation:**

A 41-year-old female with a complex medical history including MS presented with severe desquamation and erythematous skin lesions on the hands, feet, and legs following treatment with secukinumab for psoriatic arthritis that necessitated discontinuation of secukinumab. A biopsy suggested drug eruption, with a differential diagnosis including palmoplantar pustular psoriasis. After multidisciplinary consultation, treatment was switched to guselkumab, an IL-23 inhibitor, which resulted in clinical improvement.

**Conclusion:**

This case highlights the potential for paradoxical reactions to IL-17 inhibitors and the efficacy of IL-23 inhibitors as an alternative therapy. Multidisciplinary approach was crucial for optimal patient management.

## INTRODUCTION

Secukinumab is an IL-17A inhibitor, i.e., a critical cytokine in the pathogenesis of psoriasis and PsA.^1,3^ It has demonstrated considerable efficacy in mitigating both skin and joint symptoms in affected individuals.1 However, rare adverse events, including paradoxical psoriasis/paradoxical eczema have been reported, in which patients experience either the emergence of new psoriatic lesions or worsening of pre-existing ones.^2,4^

Guselkumab, an IL-23 inhibitor, that demonstrated efficacy in patients intolerant to or inadequately managed with other treatments, including TNF or IL-17 inhibitors.^5^ It has shown significant efficacy in addressing both dermatologic and joint manifestations of PsA.^5^ Notably, guselkumab generally exhibits a more favourable side-effect profile compared to anti TNF inhibitors,^6^ although ongoing studies are essential to comprehensively understand its long-term safety.

This report presents a unique case of sever pustular palmoplantar psoriasis induced by secukinumab in a patient of psoriatic arthritis, successfully managed with guselkumab.

## CASE REPORT

**Figure 1.**
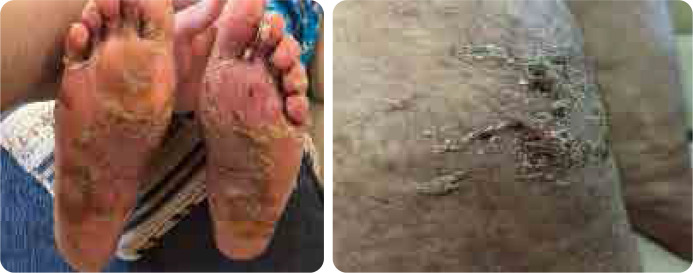
Skin peeling of planter surface of feet, scaly skin rash on the legs.

**Figure 2.**
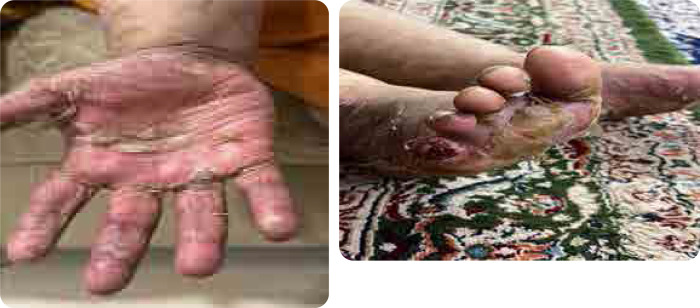
Peeling of the skin of hand and feet with ulcerations.

**Figure 3.**
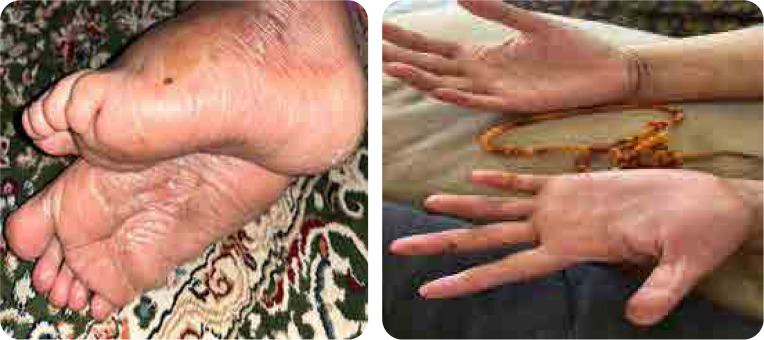
Improvement of palmoplantar skin after treatment with guselkumab.

Our patient is a 41-year-old female with a 15- year history of multiple sclerosis (MS), controlled on azathioprine 200 mg daily. She presented with multiple scaly scalp plaques, periauricular scaly rash and fingernails dystrophy, associated with interphalangeal and wrist joints arthritis. A skin biopsy of the affected areas confirmed dermal fibrosis with vascular proliferation, with no signs of vasculitis. The patient was diagnosed as psoriatic arthritis and psoriasis. She was initiated on secukinumab 300 mg weekly for 4 weeks then monthly. After two months, the patient experienced severe peeling and scaling on both hands and feet, new erythematous plaques on her legs, with persistent pain and desquamation, significantly impairing quality of life (**Figure 1** and **Figure 2**), topical corticosteroids- clobetasol propionate ointment for hands and feet and mometasone ointments for other body parts were used with limited improvement. A punch biopsy from the palmer surface of the right hand which had the most prominent and active lesion revealed spongiotic dermatitis, suggestive of a drug eruption. Following a multidisciplinary discussion between rheumatology and dermatology, with a clinical diagnosis of palmoplantar pustulosis deciding switch treatment to guselkumab to control both skin and psoriatic arthritis. Upon switching, the patient reported significant improvement in her symptoms with resolution of peeling, erythema, and pain (**Figure 3**).

## DISCUSSION

Our case highlights the challenges of managing psoriatic arthritis in a patient with other autoimmune diseases, such as MS.^7^ Secukinumab is widely used to treat moderate-to-severe plaque psoriasis and psoriatic arthritis due to its significant efficacy in alleviating both skin lesions and joint symptoms.^1,3^ However, it can paradoxically exacerbate psoriasis or trigger new dermatologic conditions, as seen in this patient.^2,4^

One of the unintended consequences of IL17 inhibition is a shift from a Th17 to a Th2-dominated immune response, which may explain why some patients develop paradoxical reactions such as eczema-like eruptions or flares of psoriasis. These reactions often occur weeks to months after starting IL-17 inhibitors.^8^

In our case, there were some debates about the diagnosis as the clinical features were initially suggestive of a psoriasiform eruption with palmoplantar vesiculation and nail involvement which were more compatible with diagnosis of palmoplantar pustulosis as a localised type of pustular psoriasis while the histopathology revealed spongiotic changes, which are more consistent with an eczematous process. On full-body examination, there were no features suggestive of classic eczema such as flexural involvement or widespread xerosis. We thus overweighed the diagnosis of palmoplantar pustulosis over a possible differential of paradoxical eczema. The development of severe palmoplantar psoriasis/paradoxical eczema following secukinumab initiation led to its discontinuation and necessitated a switch to an alternative therapy after a weak response to local steroids.

The switch to guselkumab resulted in significant improvement in our patient’s symptoms, particularly in reducing the peeling and pain in her hands and feet.^6^

In mild and localised cases, it is usually safe to continue the biologic while adding topical corticosteroids, calcineurin inhibitors (like tacrolimus), and emollients to control symptoms. However, if the reaction is more widespread or severe, temporarily stopping the biologic may be necessary. During this time, short courses of systemic treatments such as oral steroids or immunosuppressants like cyclosporine can help manage the flare. If the same reaction comes back after restarting the biologic, switching to a different class—especially IL-23 inhibitors like guselkumab—may be more appropriate.^9^

Collaboration between dermatology, rheumatology, and neurology was essential to adjust the treatment plan effectively, balancing the need to control psoriatic arthritis without compromising the patient’s neurologic stability. Early recognition of drug-induced exacerbation allowed for timely intervention, improving the patient’s symptoms and quality of life.^4^

Our case further highlights the need for individualised treatment approaches and close monitoring of biologic therapies, particularly in patients with multiple immune-mediated diseases.^7^ The successful transition to guselkumab demonstrates its potential as an effective and well-tolerated option for managing psoriatic arthritis in complex cases.^5^

## CONCLUSION

This case underscores the importance of early recognition of adverse reactions to biologics, individualised treatment adjustments in patients with complex medical histories, and close collaboration among specialists to ensure optimal care. The successful transition to guselkumab offers a promising alternative for this patient, aiming to control her psoriatic arthritis without compromising her neurologic stability.

## ETHICS APPROVAL AND CONSENT TO PARTICIPATE

The case report was approved by the local ethics committee in KFMC.

## AVAILABILITY OF THE DATA AND MATERIAL

All data and materials are available when requested

## COMPETING INTERESTS

The authors declare that they have no known competing interests or personal relationships that could have appeared to influence the work reported in this case report.

## FUNDING

This research did not receive any specific grant from funding agencies in the public, commercial, or not-for-profit sectors.
